# Ensemble coding of context-dependent fear memory in the amygdala

**DOI:** 10.3389/fnbeh.2013.00199

**Published:** 2013-12-13

**Authors:** Caitlin A. Orsini, Chen Yan, Stephen Maren

**Affiliations:** ^1^Department of Psychology, University of MichiganAnn Arbor, MI, USA; ^2^Program in Neurosciences and Mental Health, Hospital for Sick ChildrenToronto, ON, Canada; ^3^Department of Neuroscience Program, University of MichiganAnn Arbor, MI, USA; ^4^Department of Psychology and Institute for Neuroscience, Texas A&M University, College StationTX, USA

**Keywords:** amygdala, prefrontal cortex, hippocampus, *Arc*, renewal, context, extinction, fear

## Abstract

After fear conditioning, presenting the conditioned stimulus (CS) alone yields a context-specific extinction memory; fear is suppressed in the extinction context, but renews in any other context. The context-dependence of extinction is mediated by a brain circuit consisting of the hippocampus, prefrontal cortex (PFC) and amygdala. In the present work, we sought to determine at what level of this circuit context-dependent representations of the CS emerge. To explore this question, we used cellular compartment analysis of temporal activity by fluorescent *in situ* hybridization (catFISH). This method exploits the intracellular expression profile of the immediate early gene (IEG), *Arc*, to visualize neuronal activation patterns to two different behavioral experiences. Rats were fear conditioned in one context and extinguished in another; 24 h later, they were sequentially exposed to the CS in the extinction context and another context. Control rats were also tested in each context, but were never extinguished. We assessed *Arc* mRNA expression within the basal amygdala (BA), lateral amygdala (LA), ventral hippocampus (VH), prelimbic cortex (PL) and infralimbic cortex (IL). We observed that the sequential retention tests induced context-dependent patterns of *Arc* expression in the BA, LA, and IL of extinguished rats; this was not observed in non-extinguished controls. In general, non-extinguished animals had proportionately greater numbers of non-selective (double-labeled) neurons than extinguished animals. Collectively, these findings suggest that extinction learning results in pattern separation, particularly within the BA, in which unique neuronal ensembles represent fear memories after extinction.

## Introduction

The extinction of conditioned fear has direct parallels with cognitive-behavioral treatments, such as exposure therapy, for anxiety disorders in humans (Bouton, 1988; Zinbarg et al., 1992; Rothbaum and Schwartz, 2002; Mineka and Oehlberg, 2008). During extinction, a previously conditioned stimulus (CS) is repeatedly presented without the unconditioned stimulus (US; Pavlov, 1927), resulting in a decrease in learned fear behavior, often measured as freezing. Importantly, extinction does not erase the original fear memory; instead, it creates an inhibitory memory that suppresses fear (Pavlov, 1927; Bouton, 1993; Maren, 2011). The inhibitory memory is context-dependent insofar as fear will renew if the CS is presented in a different context (Bouton and Bolles, 1979). This indicates that contextual cues modulate the retrieval of extinguished fear memories, leading to the expression or suppression of fear to the CS in the renewal or extinction contexts, respectively.

Contextual regulation of extinction is mediated by a brain circuit involving the hippocampus, prefrontal cortex (PFC) and amygdala (Maren and Quirk, 2004; Herry et al., 2008; Maren, 2011; Orsini et al., 2011). For example, the renewal of fear involves hippocampal projections to both PFC and the basal amygdala (BA). Inactivation of the hippocampus disrupts context-dependent firing in the amygdala (Maren and Hobin, 2007) and the elimination of hippocampal input to the BA either directly or indirectly via the prelimbic area (PL) of the PFC disrupts renewal (Orsini et al., 2011). Moreover, fear renewal is associated with *Fos* expression in PL and BA, whereas fear suppression yields *Fos* expression in the infralimbic cortex (IL) of the PFC and inhibitory intercalated cells (ITC) in the amygdala (Knapska and Maren, 2009). These findings suggest a circuit model in which structures upstream of the BA, including the hippocampus and PFC, sculpt its activity to produce context-dependent fear behavior.

Though the circuitry underlying the contextual modulation of fear is fairly well understood, it is less clear how neuronal networks in the hippocampus, PFC, and amygdala represent extinguished CSs. Neurophysiological recordings in the BA suggest that two separate populations of CS-responsive neurons are engaged during either the renewal or suppression of fear (Herry et al., 2008). However, it is not known whether similar cell assemblies exist in the PFC and hippocampus and whether these brain areas differ with regards to the number of neurons responding in a context-dependent manner. To address these questions, the present study used cellular compartment analysis of temporal activity by fluorescent *in situ* hybridization (catFISH) to visualize neuronal activation to two different behavioral experiences (Guzowski and Worley, 2001). Here, we use the cellular distribution of *Arc* mRNA to characterize neuronal ensembles in the PFC, hippocampus and amygdala that are engaged during the retrieval of fear and extinction memories.

## Materials and Methods

### Subjects

Experimental subjects were male Long-Evans rats (200–224 g; Blue Spruce) obtained from a commercial supplier (Harlan Sprague-Dawley, Indianapolis, IN). Rats were housed individually in clear plastic hanging cages and were maintained on a 14:10 light:dark cycle with access to food and water *ad libitum*. Prior to the start of the experiment, rats were handled 15–20 s/day for 5 continuous days so as to acclimate the animals to the experimenter. All experimental procedures were carried out in accordance with the protocols approved by the University of Michigan Committee on the Use and Care of Animals (UCUCA).

### Behavioral apparatus

All behavioral sessions occurred in eight identical observation chambers (30 × 24 × 21 cm; Med-Associates, St. Albans, VT), constructed of a Plexiglas ceiling, back and door and two aluminum sidewalls. The floor of each observation chamber consisted of 19 stainless steel rods (4 mm in diameter) by which the footshock US was delivered. The rods of the floor were wired to a shock source and a solid-state shock scrambler (Med-Associates, St. Albans, VT). Within each observation chamber, a speaker was mounted on one sidewall to deliver the acoustic CS. Lastly, each chamber contained a house light and ventilation fans that could be manipulated to create distinct contexts in the experimental. Importantly, each observation chamber was situated within a sound-attenuating cabinet.

A three-context (“ABC/ACB”) renewal design was used in this experiment. For Context A (fear conditioning context), room lights, house lights and ventilation fans (65 dB) were turned on and the cabinet doors were left open. Each observation chamber was cleaned with 1% acetic acid. In Context B (extinction and test context), house lights and ventilations fans were turned off and the cabinet doors were closed. Chambers were cleaned with 1% ammonium hydroxide and the room was illuminated by fluorescent red lights. For Context C (extinction and test context), ventilation fans were left off, but the house lights were turned on. The cabinet doors were left open and the room was lit by fluorescent red lights. Chambers were cleaned with 10% ethanol and black Plexiglas floors were put on top of the grids in each chamber. For each context, stainless steel plans containing a thin layer of the context’s respective odor were inserted below the grid floor of each observation chamber.

In each behavioral session, motor activity was measured by recording the displacement of each chamber by a load cell platform located beneath each chamber. Before the experiment commenced, each load cell amplifier was calibrated to a fixed chamber displacement and the output of each amplifier was set to a gain that optimally detected freezing behavior (vernier dial = 8; somatomotor immobility except that necessitated for breathing). The output of each load cell amplifier (−10 to +10 V) was subsequently digitized (5 Hz), resulting in one observation per rat every 200 ms (300 observations per rat per min), and acquired online using Threshold Activity software (Med-Associates, St. Albans, VT). The absolute values of the load cell voltages were multiplied by 10, yielding an activity score that ranged from 0 to 100. If at least five continuous load-cell values (or at least 1 second’s worth) fell below the freezing threshold (threshold = 10), freezing was scored for that time period. This method of assessing freezing behavior has been used previously and is tightly correlated with time sampling of freezing behavior by trained observers (Maren, 1998). In all behavioral sessions, freezing was assessed during the pre-trial, or baseline, period and during all subsequent trials, each of which consisted of a CS presentation and the interstimulus interval (ISI).

### Behavioral procedures

**Figure 1:**
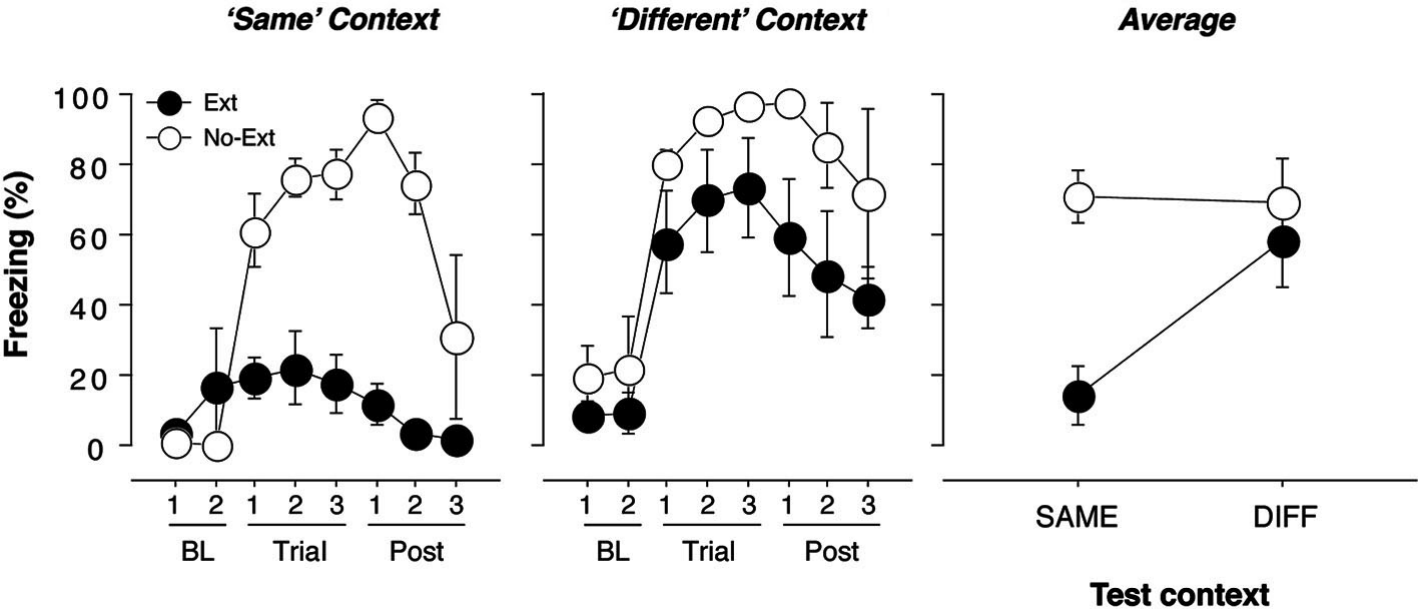
**Freezing behavior in extinguished and non-extinguished rats during the test sessions.** Rats were fear conditioned in Context A on Day 1 and subsequently underwent context exposure and extinction in Context C and Context B (contexts were counterbalanced) on Day 2. During the retrieval test on Day 3, rats were tested twice: once in the extinction context and once outside the extinction context, with each test separated by an 18-min interval. Each test consisted of three tone-alone presentations with 30 s ISIs. Immediately after the second test, rats were sacrificed and brains were extracted for catFISH processing. Freezing behavior during conditioning and extinction was typical and did not differ from previous reports (Orsini et al., 2011); thus, it is not displayed. **(A)** Mean percentage of freezing (±SEM) to the CS when presented within the extinction context (“Same”). Freezing was measured during the baseline (BL) period (two 1-min blocks), during three 40-s trials, each of which consisted of a 10-s CS presentation and the subsequent 30-s ISI, and during the post-tone period (Post) (three 1-min blocks). **(B)** Mean percentage of freezing (±SEM) to the CS when presented outside of the extinction context (“Different”). Freezing was measured during the baseline (BL) period (two 1-min blocks), during three 40-s trials, each of which consisted of a 10-s CS presentation and the subsequent 30-s ISI, and during the post-tone period (Post) (three 1-min blocks). **(C)** Mean percentage of freezing (±SEM) during the test trials for extinguished (EXT) and non-extinguished (NO-EXT) rats. For all figures, freezing was collapsed across Same/Diff and Diff/Same groups to yield an overall mean percentage of freezing (±SEM) for the renewal and extinction test.

Twelve rats were randomly assigned to two groups: those that received extinction training (EXT; *n* = 8) and those that did not (NO-EXT; *n* = 4). The experiment consisted of a three-context renewal design whereby animals were fear conditioned in Context A, extinguished in Context B and tested in Context B and C (Figure 1). This yielded conditions in which EXT rats were tested in the extinction context (SAME; ABB) and in another context that had not hosted extinction (DIFF; ABC). Rats in the NO-EXT group were tested in both of the test contexts. The EXT group was further subdivided into rats that received the DIFF test first and the SAME test last (D/S) and rats that received the SAME test first and the DIFF test last (S/D). Similarly, NO-EXT rats were divided into rats that were tested in Context B first and tested in Context C last (B/C) and those that were tested in Context C first and Context B last (C/B).

One week after being housed, rats underwent fear conditioning, which consisted of five tone (10 s, 85 dB, 2 kHz)-footshock (1.0 mA, 2.0 s) pairings with ISIs of 60 s. The chamber position of each rat was counterbalanced across experimental group and test order (S/D, D/S, B/C, C/B). Twenty-four hours after conditioning, EXT rats underwent extinction (45 tone-alone presentations with 30 s ISIs) in Context B. NO-EXT rats were also placed in Context B, but did not receive CS presentations. Prior to the extinction/no-extinction session, all animals were exposed to Context C. This ensured that all animals were equally familiar with all contexts involved in the experiment. Twenty-four hours after extinction, rats were returned to the observation chambers for the first of two tests. Each test session consisted of 3 tone-alone presentations with 30 s ISIs in either Context B or C. After the first test session, rats were returned to their home cage for 18 min before being tested again in the alternate context. Immediately after the last test, rats were lightly anesthetized with isofluorane and killed for brain tissue extraction. Brains were quickly extracted, flash frozen in a vial of isopentane that was immersed in dry ice and subsequently stored at −80° C until sectioning. The relative timing of this design was used to parallel the expression profile of the immediate early gene (IEG), *Arc*. Under basal conditions, *Arc* expression is very low (Guzowski et al., 2005). However, upon a behavioral experience (or any type of stimulation associated with synaptic plasticity), *Arc* mRNA can be observed in the nucleus within 5 min and in the cytoplasm within 25 min. Importantly, this allows one to assess neuronal activation induced by two temporally disparate behavioral experiences (Guzowski and Worley, 2001; Guzowski et al., 2005). As such, in the present experiment, cytoplasmic staining would correspond to the first test session and nuclear staining would correspond to the last test session. In all behavioral sessions, freezing was used as the index of fear.

### Fluorescent in situ hybridization (FISH)

Upon completion of the experiment, coronal sections (20 µm) were collected with a cryostat maintained at a constant temperature of −21° C and arranged on electrostatic slides (Histobond). Slides were stored at −80° C until FISH procedures commenced.

Digoxigenin (DIG)-labeled *Arc* riboprobes were generated using a commercial MAXIscript T7/T3 *in vitro* transcription kit (Ambion). After being treated with DNase, the riboprobes were subsequently purified using Mini Quick Spin RNA Columns (Roche). Successful yield of the DIG-labeled riboprobe was confirmed by a gel electrophoresis; purity and concentration was assessed on a NanoDrop. The riboprobe was then stored at −80° C until use.

For FISH procedures, slide-mounted sections were first thawed to room temperature (RT) and were then fixed in 4% buffered paraformaldehyde for 10 min. After a wash in a 2X saline-sodium citrate buffer (SSC), sections were treated with acetic anhydride/triethanolamine and then incubated in a 1:1 acetone/methanol mix. Following another 2X SSC wash, 200 µl of 1X pre-hybridization buffer (Sigma) was applied to each slide and coverslips were overlaid. Slides were incubated in a humid chamber for 30 min at RT after which, 150 µl of 1X hybridization buffer containing the DIG-labeled riboprobe (100 ng) was applied to each slide. Slides with overlying coverslips were incubated in a humid chamber overnight at 56° C. Twenty-four hours later, slides were washed several times in 2X SSC buffer and then treated with RNase (1:1000; diluted in 2X SSC) for 30 min at 37° C. Slide-mounted sections were then washed in several stringent SSC washes, two of which were 0.5X SSC at 56° C. After the final SSC wash, slides were incubated in a 1% H_2_O_2_ solution for 30 min, quenching any endogenous peroxidase activity in the tissue. After two separate 2X SSC washes, slides were introduced to a tris-buffered solution (TBS) for 5 min, followed by the application of 150 µl of blocking buffer [Normal Donkey Serum (NDS; JacksonImmuno) mixed with blocking reagent (Roche)] to each slide. Slides were incubated with overlying coverslips in the blocking buffer for 30 min in a humid chamber at RT, after which 150 µl of the primary antibody solution [mouse anti-DIG conjugated to horseradish peroxidase (HRP; JacksonImmuno) at 1:300; diluted in blocking buffer without NDS] was applied to each slide and coverslips were placed on each slide. Slides were incubated in a humid chamber for 2 h at RT and subsequently washed several times in TBS with 0.05% Tween-20 (TBS-T). To amplify the *Arc* signal, 100 µl of tyramide-signal amplification (TSA)-biotin conjugated solution was applied. Coverslips were overlaid, and slides were incubated in a humid chamber for 30 min at RT. Slides were subsequently washed twice in TBS-T and once in TBS. To detect *Arc* mRNA and stain neuronal nuclei, streptavidin conjugated to AlexaFluor 488 (1:300; Invitrogen) and Hoechst (1:500; Sigma), respectively, were diluted in TBS and 150 µl of this solution was added to each slide. Coverslips were overlaid and slides were incubated for 1.5 h at RT. Finally, slides were washed twice in TBS-T, once in TBS and coverslipped with Vectashield mounting medium (Vector Labs; without DAPI).

### Confocal microscopy and image analysis

Stained sections were imaged on an Olympus Fluoview (FV1000) confocal microscope equipped with six lasers (405, 458, 488, 515, 561 and 633 nm lasers). Images were collected using an Olympus 40X/1.30 oil immersion lens and each image was *z*-sectioned in 0.5 µm optical sections. Six images were analyzed for each brain region [the PL, IL, VH, BA and lateral amygdala (LA)] for each rat, similar to other published catFISH studies (Han et al., 2007, 2009). Using the publicly available ImageJ software, cells were characterized as one of the following: nuclear, cytoplasmic, or nuclear/cytoplasmic. Importantly, only those cells that (1) were not cut off on the edges of the image, and (2) were present throughout the entire *z*-stack were included in the analyses. Cells were denoted as “nuclear” if they showed one or two robust foci with high levels of saturation that were restricted only to the nucleus. Neurons were denoted as “cytoplasmic” if they showed a “halo” of *Arc* staining around the nucleus and/or diffuse perinuclear staining present across multiple sections. The nuclear/cytoplasmic (N/C; double-labeled) designation was given to cells that showed both of the aforementioned properties. Cells were counted by an investigator blind to each rat’s experimental condition. Cell counts were averaged across the samples from each region of interest; group differences in cell counts were analyzed with an ANOVA and Fisher’s protected least significant difference (PLSD) post hoc tests. Results are represented as means (±*SEM*).

### Behavioral data analysis

During all behavioral sessions, freezing was measured during the pre-CS “baseline” periods, CS presentations and ISIs. Freezing was then analyzed and reported for each trial, which consisted of a CS presentation and its subsequent ISI. For each trial, the percentage of total observations in which freezing occurred was calculated and these values were submitted to analysis of variance (ANOVA). If a significant omnibus *F*-ratio was obtained, Fisher’s PLSD post-hoc tests were performed. All data are represented as means (±*SEM*).

## Results

### Behavior

The conditioning and extinction of fear were typical, and similar to that reported using identical parameters in an earlier experiment (Orsini et al., 2011); therefore, these data are not shown. Twenty-four hours after extinction, the rats received two independent test sessions in which the CS was presented three times in both the extinction (SAME) and renewal (DIFF) contexts; these sessions were separated by 18 min and the order of the sessions was counterbalanced. As we have previously reported, extinguished rats displayed significantly lower levels of fear than non-extinguished rats when tested in the extinction context [Figure 1; *F*(1, 7) = 28.3, *p* = 0.01]. In contrast, there were no significant differences in freezing between extinguished and non-extinguished rats when tested in the renewal context [*F*(1, 7) = 3.0, *p* > 0.05]. These effects were further confirmed by a significant test (SAME/DIFF) by group (EXT/NO-EXT) interaction using an ANOVA [*F*(1, 7) = 11.7, *p* = 0.01]. Importantly, there was no effect of test order in either extinguished [*F*(1, 3) < 1] or non-extinguished [*F*(1.2) < 1] rats.

### *Arc* expression in the prefrontal cortex, ventral hippocampus and amygdala during contextual retrieval of fear after extinction

Immediately after the second retrieval test, rats were killed for brain extraction and tissue processing. We analyzed *Arc* staining in the mPFC (IL and PL), ventral hippocampus (VH), and basolateral amygdala (BLA; LA and BA analyzed separately)—all regions that we have previously implicated in fear renewal using *Fos* immunohistochemistry (Knapska and Maren, 2009). Within each brain region, the number of cells expressing cytoplasmic, nuclear, or cytoplasmic + nuclear *Arc* was counted. Depending on an individual’s test order, the cellular localization of *Arc* staining determined whether a cell was active during the retrieval test in the extinction context (SAME), the renewal context (DIFF), or both tests (BOTH) in animals that had undergone extinction (EXT). We refer to *Arc* cells that were active in only one of the retrieval tests as *context-selective* cells. For cells in non-extinguished animals (NO-EXT), SAME and DIFF refer to the matched physical contexts in which the EXT group was tested. Finally, because cell activation due to CS presentation was the main focus of this study, cells without *Arc* staining were not included in our analyses.

**Figure 2:**
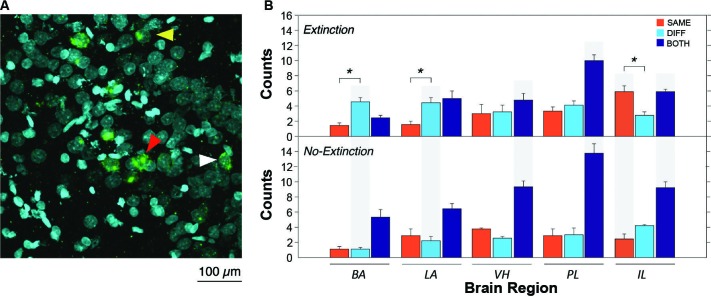
**Representative catFISH staining and subsequent cell count results. (A)** Representative confocal images VH taken at 40X magnification. White arrowheads indicate cytoplasmic staining and yellow arrowheads indicate nuclear staining. Red arrowheads indicate nuclear and cytoplasmic staining (non-selective neurons). **(B)** Raw counts of cells expressing *Arc* after in the extinction context (SAME), the renewal context (DIFF) or both contexts (BOTH). For the no-extinction group (NO-EXT), SAME and DIFF refer to the matched physical contexts in which the extinction group (EXT) was tested, because there was no SAME/DIFF relationship between the CS and test context in NO-EXT animals. Asterisks refer to significant within region differences in the number of neurons expressing *Arc* (*p* < 0.05). Gray shading indicates significant between group differences in *Arc* expression between EXT and NO-EXT groups (comparing top to bottom; *p* < 0.05). Cell counts are represented as means (±SEM) for the BA, LA, VH, prelimbic cortex (PL), and IL.

After extinction, CS presentation yielded robust *Arc* expression in the VH, mPFC, BA and LA. A representative photomicrograph of hippocampal staining is shown in Figure 2A. Importantly, the pattern of *Arc* expression differed among brain regions and was strongly influenced by extinction (Figure 2B). Inspection of the results revealed that the number of *Arc* positive cells differed amongst brain areas; specifically, the distribution of single and double-labeled cells differed across brain areas and was modulated by extinction. Indeed, extinction appeared to yield increases in the number of single-labeled cells (SAME or DIFF) in some brain areas, as well as decreasing the number of double-labeled cells (BOTH). These impressions were confirmed in a three-way ANOVA with group (EXT and NO-EXT) as a between-subjects factor and brain region (BA, LA, VH, PL and IL) and cell type (SAME, DIFF, and BOTH as within-subjects factors. This analysis yielded main effects of region [*F*(4, 28) = 2.8, *p* < 0.05] and cell type [*F*(2, 14) = 93.5, *p* < 0.0001]. In addition, there were significant two-way interactions of cell type with both group [*F*(2, 14) = 22.5, *p* < 0.0001] and brain region [*F*(8, 56) = 8.3, *p* < 0.0001] as well as a significant three-way interaction between region, group and cell type [*F*(8, 56) = 2.8, *p* < 0.05].

As shown in Figure 2B (asterisks), animals undergoing extinction exhibited different degrees of context-specific *Arc* expression in the extinction and renewal contexts in the BA, LA, and IL. In the LA and BA, post-hoc comparisons (Fisher’s LSD) revealed that there were greater numbers of *Arc* cells after CS exposure in the renewal context (when fear relapsed), compared to the extinction context (*p* < 0.05); the converse was true in IL (*p* < 0.05). These results parallel earlier observations using *Fos* as a marker of cellular activity (Knapska and Maren, 2009; Orsini et al., 2011). However, in contrast to these earlier reports, we did not observe differential activity in PL and VH, which had previously been found to show greater levels of *Fos* expression in animals tested in the renewal context. The level of *Arc* expression in non-extinguished animals was similar in the two retrieval contexts, and was generally lower than that in extinguished animals. These within-subject changes in *Arc* expression were paralleled by between-subject differences across extinguished and non-extinguished rats. Post-hoc comparisons (Fisher’s LSD, *p* < 0.05) revealed that extinction training decreased the number of double-labeled cells in the VH, PL and IL, while increasing the number of single-labeled neurons in the BA and LA (DIFF condition) and IL (SAME condition).

**Figure 3:**
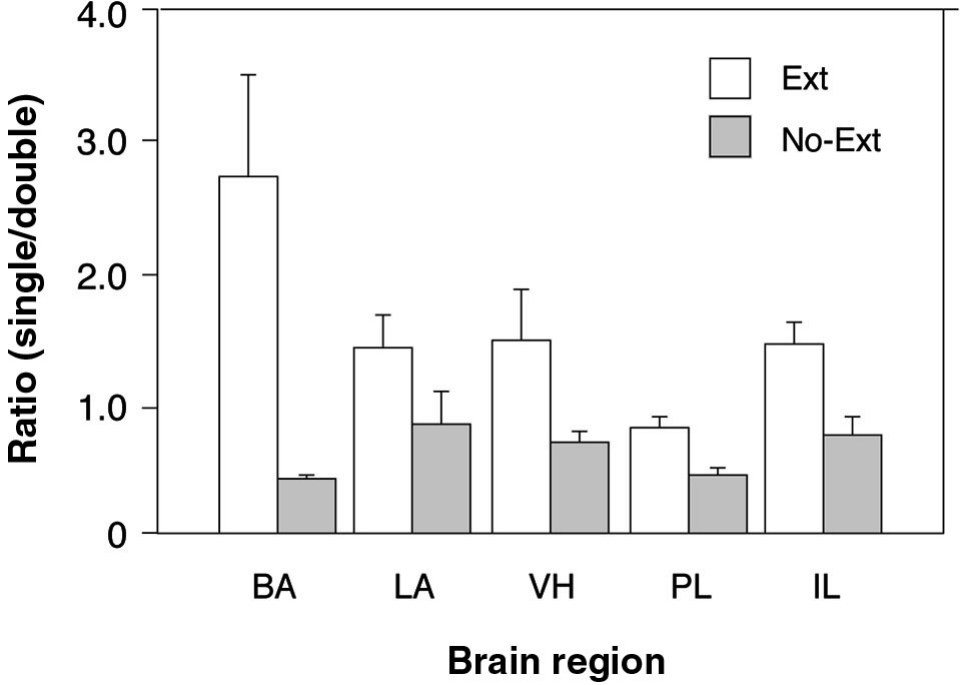
**Comparison of the ratio of context-selective to non-selective neurons between extinguished (EXT) and non-extinguished rats (NO-EXT).** Ratios are represented as means (± SEM) for the BA, LA, VH, prelimbic cortex (PL), and IL.

To further examine the possibility that extinction training increased the number of context-selective neurons in a region-specific manner (an outcome indicated by the three-way interaction in the ANOVA), we calculated the ratio of single-labeled to double-labeled neurons in each brain area by summing the number of (single-labeled) cells active only in one test context and dividing that sum by the number of (double-labeled) cells active in both. This ratio provides an index of the number of context-selective cell assemblies in each area relative to non-selective cells that were active in either retrieval context. As shown in Figure 3, there were considerable regional differences in the ratio of context-selective and non-selective neurons, and these ratios were influenced by extinction training. Specifically, extinction training increased the ratio of context-selective cells, although this effect varied by brain area. These impressions were confirmed in a two-way ANOVA with group (EXT and NO-EXT) as a between-subjects factor and brain region (BA, LA, VH, PL, and IL) as a within-subjects factor for the selectivity ratios. This analysis revealed significant main effects of group [*F*(1, 7) = 7.7, *p* < 0.05] and region [*F*(4, 28) = 3.3, *p* < 0.05], as well as an interaction between the two [*F*(4, 28) = 4.3, *p* < 0.01]. Specifically, post-hoc comparisons (Fisher’s LSD) revealed that extinction resulted in greater levels of context-selectivity in the BA, PL and IL (*p* < 0.05). Interestingly, this effect was most pronounced in BA. Within the BA, extinguished rats exhibited nearly three times the number of selective cells compared to non-selective cells, whereas non-extinguished rats exhibited the inverse pattern, exhibiting roughly twice the number of non-selective neurons. Although both LA and VH exhibited a similar trend, this was not statistically reliable. Collectively, these data suggest that extinction training contributes to the emergence of distinct cell ensembles that are recruited in a context-dependent manner in all brain regions.

## Discussion

Context-dependent retrieval of fear involves a neural circuit that interconnects the VH, BA and PFC (Maren and Quirk, 2004; Maren, 2011; Orsini and Maren, 2012). The present study used the cellular distribution of the IEG *Arc* to determine whether extinction establishes unique cell assemblies in these regions and whether memory retrieval engages these assemblies during the renewal and suppression of fear. We found that presentation of an extinguished CS in the extinction context increased *Arc* expression in the IL, whereas presentation of the same CS (in the same animal) outside the extinction context increased *Arc* expression in the BLA. In contrast to our previous reports using *Fos* to index neuronal activity (Knapska and Maren, 2009; Orsini et al., 2011), we did not observe differential *Arc* expression in the VH and PL during fear renewal. Across all brain regions, extinction increased the number of context-selective (single-labeled neurons), and this effect was particularly pronounced in the BA. Overall, these data show that extinction alters the cellular representation of fear and extinction memories, resulting in the emergence of ensembles of context-selective neurons in several brain areas, particularly the amygdala.

The present results are consistent with earlier reports showing increases in *Fos* expression in the BA and IL in response to a CS presented in the extinction context (Knapska and Maren, 2009; Knapska et al., 2012). Others have also shown that IL neurons are selectively activated during extinction recall. For example, cells in the IL exhibit an increase in burst firing after extinction (Santini et al., 2008; Chang et al., 2010). However, we did not observe differential regulation of VH or PL *Arc* expression during fear renewal and suppression. We predicted increases in VH and PL *Arc* expression in the renewal context based on our previous *Fos* work (Knapska and Maren, 2009; Orsini et al., 2011). There are at least two possible explanations to account for this discrepancy. First, in the present work, we used a within-subjects design in which the extinguished CS was presented in both the extinction and renewal contexts with a relatively short delay between each test. Although there was strong evidence for contextual modulation of behavior, including the renewal of conditional freezing outside the extinction context, it remains possible that this particular within-subjects design decreased discriminability of the contexts. Indeed, within-subjects procedures for quantifying extinction and renewal tend to yield weaker renewal effects (Hobin et al., 2003). This may have dampened the renewal-related neural activity in the VH and PL. This seems unlikely, however, given that the amygdala and IL exhibited the sort of CS-induced IEG expression we have observed in the past. Another possibility is that *Fos* and *Arc* are regulated differently, or are expressed in different populations of neurons in the brain areas examined in this study (Kubik et al., 2007). Different expression profiles of the two IEGs might account for the absence of *Arc* expression in neurons that express *Fos* during renewal in other studies.

A major finding in the present study is that extinction training increased the context-selectivity of *Arc* expression among neurons in the amygdala and mPFC, but surprisingly, not the VH. Greater context-selectivity in these brain areas was reflected in a much greater proportion of single-labeled cells in extinguished animals compared to non-extinguished animals. The emergence of distinct neuronal ensembles that represent the same auditory CS in a context-dependent manner has also been observed in electrophysiological studies. For example, single-unit recordings in the basal (Herry et al., 2008) and lateral (Hobin et al., 2003; Maren and Hobin, 2007) amygdaloid nuclei have revealed the existence of context-dependent neuronal ensembles. In these studies, neurons were found to fire preferentially to CSs either during fear renewal (“fear neurons”) or fear suppression (“extinction neurons”; Herry et al., 2008). Using cellular imaging methods that capture a broader sample of neuronal activity than single-unit recordings, we now show that context-selective neuronal ensembles are particularly prominent in the BA, but also present in the mPFC. It should be noted that we cannot exclude the possibility that the contexts alone may have contributed to the patterns of *Arc* expression we observed. That is, the extinction context may have acquired inhibitory properties that resulted in differential *Arc* expression relative to the context that never experienced extinction. However, earlier work has shown that context-specific single-unit activity in the amygdala is expressed when the extinction history of test contexts are equated, suggesting that this factor is unlikely to account for the patterns of *Arc* expression we observed (Hobin et al., 2003). These results support the view that extinction training yields a unique memory that is different from the original fear memory (Pavlov, 1927; Bouton, 1993; Quirk and Mueller, 2008).

Considerable research has shown that the expression of fear responses after extinction is dependent on the context in which the CS is presented; this enables an organism to discriminate between different meanings of the same stimulus (Bouton, 2004; Orsini and Maren, 2012). As such, representations of the CS-context associations learned during conditioning and extinction must be encoded in different neuronal populations. If not, CS-“no US” memories learned during extinction would compete with the CS-US memories learned during conditioning and interfere with the performance of fear in all contexts. One process that might enable context-dependent representations of fear and extinction memories is *pattern separation*, which is mediated by the hippocampus (Shapiro and Olton, 1994; Treves and Rolls, 1994; Yassa and Stark, 2011). Interestingly, the present data suggest that pattern separation (at least as it is instantiated in *Arc*-positive neuronal ensembles) for fear and extinction memories may be mediated by the amygdala and mPFC. Indeed, the possibility that the amygdala plays a role in pattern separation has been previously suggested in studies of reward processing (Gilbert and Kesner, 2002). As such, it is possible that pattern separation processes in the amygdala are important for discriminating associations between a CS and the presence and absence of biologically-relevant stimuli.

Surprisingly, we did not observe a significant emergence of context-selective cells in the VH (although there was a trend in this direction). We expected this outcome given the large number of studies suggesting a role for the hippocampus in spatial pattern separation (Shapiro and Olton, 1994; Treves and Rolls, 1994; Yassa and Stark, 2011). One possibility is that neuronal ensembles within the hippocampus represent both the relationship between the CS and the context in which it is retrieved, as well as CS memories that are not defined by context (at least the renewal and extinction contexts). As described above, this is consistent with the process of separation whereby similar representations of the CS are stored in non-overlapping cellular populations to prevent interference during memory retrieval. The diverse representations of the CS endow the VH with the ability to promote the flexible and context-dependent expression of behavior mediated by the amygdala. Importantly, the context-dependence of amygdala neuronal activity is unlikely to be the mere reflection of contextual processing in hippocampal and cortical afferents. One possibility is that the context-dependence of amygdala activity emerges locally from an interaction of hippocampal and cortical afferents.

We have recently a proposed a neuroanatomical model by which contextual information regulates fear behavior after extinction (Orsini et al., 2011). Specifically, we have suggested that context-dependent retrieval of fear requires convergent input in the BA from the VH and PL. This is supported by the fact that BA-projecting neurons in the VH and PL are engaged during renewal and that the disruption of communication between the VH and PL or BA impairs renewal. Moreover, a recent report finds that amygdala neurons active during the suppression of fear receive synaptic input from IL, whereas those active during fear renewal receive input from VH and PL (Knapska et al., 2012). The present study extends these findings by providing insight into how distinct CS representations emerge in the BA after extinction. We propose that convergent input from the PL and VH in the BA during extinction causes the appearance of segregated cell assemblies devoted to extinction recall or renewal. In support of this claim, we have previously found that the disconnection of the VH and PL or VH and BA had no effect on non-extinguished fear, but severely impaired the recovery of extinguished fear (Orsini et al., 2011). Furthermore, the present study shows that whereas fear to a non-extinguished CS is represented by overlapping populations in the BA, segregated cell assemblies emerge after extinction. Of course, these results do not indicate whether PL and VH input converge on similar neurons or how their activity actually causes these cell assemblies to emerge. Interestingly, it has been shown that the same VH neurons project to cells in both the amygdala and PL (Ishikawa and Nakamura, 2006) and that firing activity of PFC and amygdala neurons can become entrained to hippocampal theta rhythm (Pape et al., 1998; Seidenbecher et al., 2003; Jensen, 2005; Pape et al., 2005; Adhikari et al., 2010; Colgin, 2011). By this view, it is conceivable that the VH serves as an anatomical hub that promotes the synchronized activity of the circuit during extinction. This entrainment could aid in sculpting the formation of discrete populations of BA cells that are active during renewal or suppression of fear.

In conclusion, the present study provides new insight into how neuronal ensembles in the amygdala, hippocampus, and PFC participate in the context-dependent representations of extinguished CSs. The emergence of context-dependent representations of extinguished CSs among neuronal ensembles in the BA may enable a CS to either renew or suppress fear. By this view, BA “fear neurons” may be coupled to downstream fear effectors in the central medial amygdala, for example, whereas “extinction neurons” couple to inhibitory networks in the central lateral nucleus or intercalated clusters that limit fear. Future studies are clearly needed to address this possibility.

## Conflict of interest statement

The authors declare that the research was conducted in the absence of any commercial or financial relationships that could be construed as a potential conflict of interest.
